# Knockdown of long non-coding RNA HOTAIR inhibits malignant biological behaviors of human glioma cells via modulation of miR-326

**DOI:** 10.18632/oncotarget.4290

**Published:** 2015-06-27

**Authors:** Jing Ke, Yi-long Yao, Jian Zheng, Ping Wang, Yun-hui Liu, Jun Ma, Zhen Li, Xiao-bai Liu, Zhi-qing Li, Zhen-hua Wang, Yi-xue Xue

**Affiliations:** ^1^ Department of Neurobiology, College of Basic Medicine, China Medical University, Shenyang 110122, China; ^2^ Institute of Pathology and Pathophysiology, China Medical University, Shenyang 110122, China; ^3^ Department of Neurosurgery, Shengjing Hospital of China Medical University, Shenyang 110004, China; ^4^ Department of Physiology, College of Basic Medicine, China Medical University, Shenyang 110122, China

**Keywords:** lncRNA, HOTAIR, miR-326, glioma, FGF1

## Abstract

Glioma is the most common and aggressive primary adult brain tumor. Long non-coding RNAs (lncRNAs) have important roles in a variety of biological properties of cancers. Here, we elucidated the function and the possible molecular mechanisms of lncRNA HOTAIR in human glioma U87 and U251 cell lines. Quantitative RT-PCR demonstrated that HOTAIR expression was up-regulated in glioma tissues and cell lines. Knockdown of HOTAIR exerted tumor-suppressive function in glioma cells. Further, HOTAIR was confirmed to be the target of miR-326 and miR-326 mediated the tumor-suppressive effects of HOTAIR knockdown on glioma cell lines. Moreover, over-expressed miR-326 reduced the FGF1 expression which played an oncogenic role in glioma by activating PI3K/AKT and MEK 1/2 pathways. In addition, the *in vivo* studies also supported the above findings. Taken together, knockdown of HOTAIR up-regulated miR-326 expression, and further inducing the decreased expression of FGF1, these results provided a comprehensive analysis of HOTAIR-miR-326-FGF1 axis in human glioma and provided a new potential therapeutic strategy for glioma treatment.

## INTRODUCTION

Glioma is the most common and most aggressive malignant primary adult brain tumor. The cause of glioma is unknown and the median survival of patients with glioma is less than 15 months under conventional treatments [1, 2]. Thus, the effective methods for the glioma patients' treatment are urgently needed.

Long non-coding RNAs (lncRNAs) are highly conservative across mammalian species. Anomalous expression of lncRNAs have been reported in a wide variety of human diseases including cancers [3, 4]. Additionally, lncRNAs, such as HOX transcript antisense intergenic RNA (HOTAIR), metastasis-associated lung adenocarcinoma transcript 1 (MALAT1), prostate cancer associated non-coding RNA 1 (PRNCR1), prostate cancer gene expression marker 1 (PCGEM1) and H19 play critical roles in the progression of glioma [5]. However, the functions of lncRNAs in glioma are still partially understood [6, 7]. HOTAIR, a ~2.2-kb long non-coding RNA transcribed from the HOXC locus, is over-expressed in most solid cancers and is correlated with the tumor invasion, progression, metastasis and poor prognosis [8–12]. Recent report has indicated that HOTAIR primarily serves as a prognostic factor for glioma patient survival, a biomarker for identifying glioma molecular subtypes, as well as a critical regulator of cell cycle progression [13]. It could interact with polycomb repressive complex 2 (PRC2) to promote cell cycle progression in glioma [14]. These results indicated that HOTAIR might be an oncogene in glioma. However, its biological role in glioma and the underlying molecular mechanism remain undefined.

Recent studies have shown that HOTAIR could inhibit miR-7 expression, resulting in the up-regulated SETDB1 expression and promoted epithelial-mesenchymal transition (EMT) in breast cancer stem cells [15]. In gallbladder cancer, HOTAIR is a c-Myc-activated driver of malignancy, which acts in part through repression of miR-130a [16]. Moreover, HOTAIR inhibits miR-34a expression in prostate cancer [17], miR-331-3p expression in gastric cancer [18] and miR-141 expression in renal carcinoma cells [19]. Taken together, HOTAIR plays a critical role in human cancers by inhibiting miRNAs.

In the present study, we aimed to investigate the expression and function of HOTAIR in human glioma cells. Moreover, the interaction among HOTAIR, miR-326 and fibroblast growth factor 1 (FGF1) was also studied in order to reveal the underlying mechanisms. Our findings will give a new direction for the treatment of glioma patients.

## RESULTS

### Knockdown of HOTAIR inhibited the malignant behaviors of glioma cells

**Figure 1:**
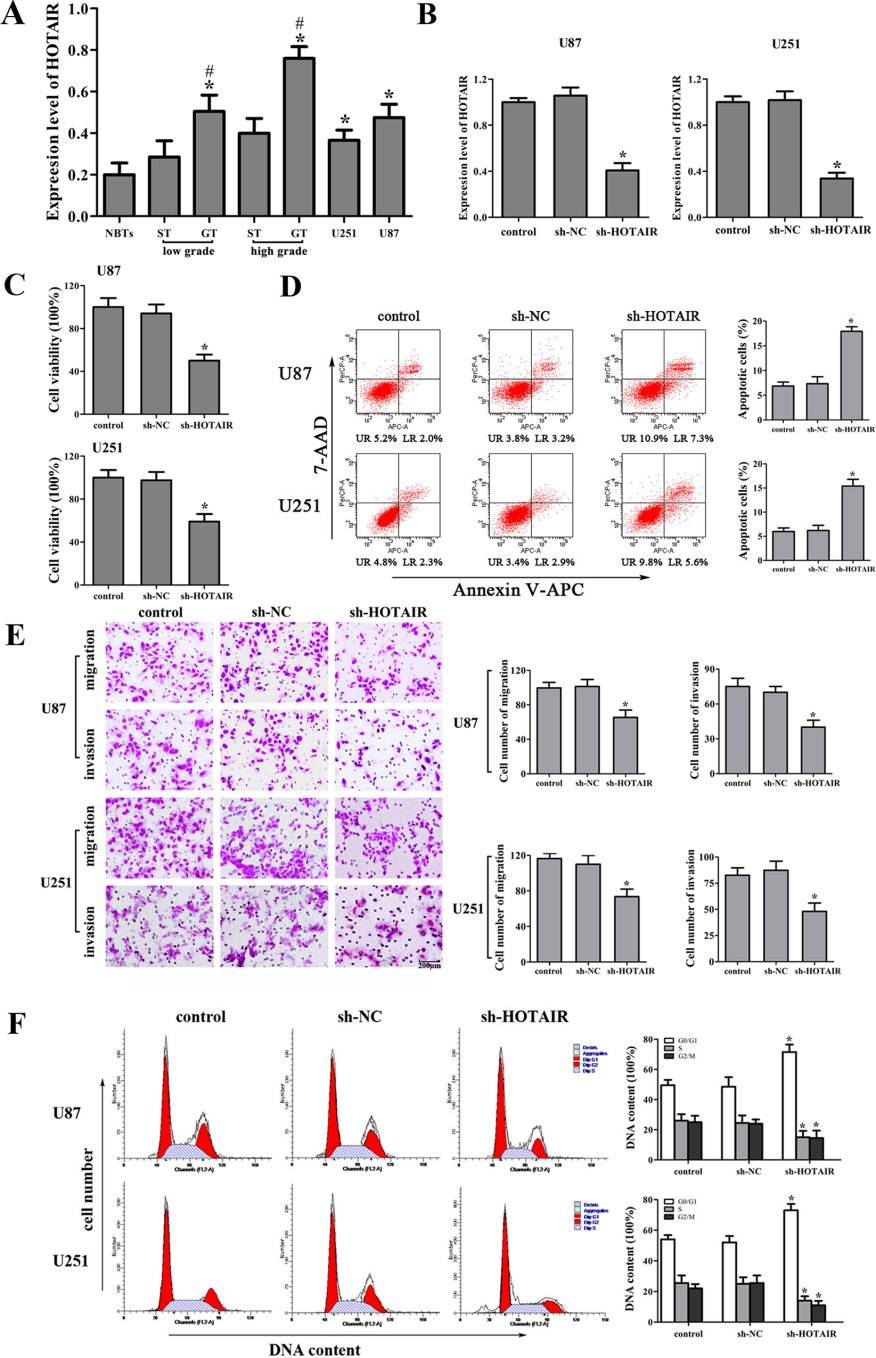
Effect of HOTAIR knockdown in human glioma cell lines **A.** HOTAIR expression in normal brain tissues (NBTs), different grades of human glioma tissues (GT), the surrounding non-neoplastic tissues (ST) and U87 and U251 glioma cell lines. Data are presented as the mean ± SD (*n* = 3, each group). *P < 0.01 vs. NBTs group. ^#^*P* < 0.05 vs. ST group. **B.** Relative expression of HOTAIR after cells transfected with short-hairpin RNA plasmids of HOTAIR. Data are presented as the mean ± SD (*n* = 5, each group). *P < 0.05 vs. sh-NC group. **C.** Effect of sh-HOTAIR on cell proliferation of U87 and U251 cells. **D.** Effect of sh-HOTAIR on apoptosis of U87 and U251 cells. **E.** Effect of sh-HOTAIR on cell migration and invasion of U87 and U251 cells. Scale bars represent 200 μm. **F.** Effect of sh-HOTAIR on cell cycle of U87 and U251 cells. Data are presented as the mean ± SD (*n* = 5, each group). *P < 0.05 vs. sh-NC group.

The expression levels of HOTAIR in human glioma tissues and cell lines were analyzed by real-time PCR. As shown in Figure 1A, HOTAIR expression was significantly up-regulated in the glioma tissues (GT) and two glioma cell lines compared with the surrounding non-neoplastic tissues (ST) and normal brain tissues (NBTs), respectively. Furthermore, HOTAIR expression was positively correlated with the histopathological grades of gliomas.

To explore the possible biological significance of HOTAIR in tumorigenesis, we next evaluated the effects of HOTAIR inhibition on the cell proliferation, apoptosis, migration, invasion and cell cycle. The stable sh-HOTAIR U87 and U251 cell lines were used in the subsequent experiments. The knockdown efficiency of sh-HOTAIR in U87 and U251 cell lines was shown in Figure 1B. Our results showed that HOTAIR knockdown inhibited the proliferation, migration and invasion, promoted the apoptosis and induced the cell cycle arrest in G0/G1 phase (Figure 1C, 1D, 1E and 1F). These results indicated that the knockdown of HOTAIR exerted tumor-suppressive effects in human glioma cells.

### HOTAIR was the target of miR-326

**Figure 2:**
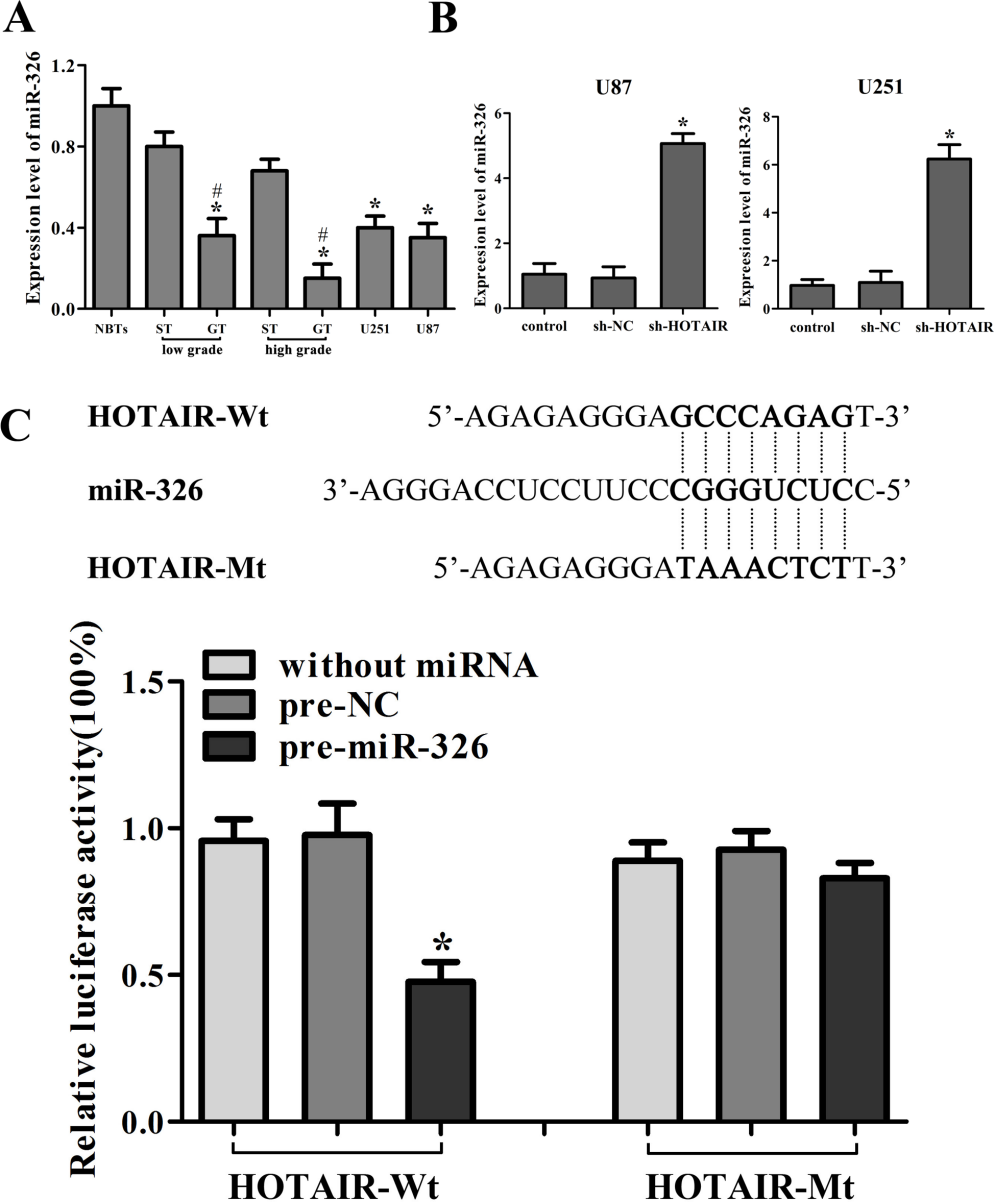
HOTAIR was the target of miR-326 **A.** MiR-326 expression in normal brain tissues (NBTs), different grades of human glioma tissues (GT), the surrounding non-neoplastic tissues (ST) and human glioma cell lines. Data are presented as the mean ± SD (*n* = 5, each group). *P < 0.01 vs. NBTs group. ^#^*P* < 0.05 vs. ST group. **B.** Relative expression of miR-326 after cells transfected with short-hairpin RNA plasmids of HOTAIR. Data are presented as the mean ± SD (*n* = 5, each group). *P < 0.05 vs. sh-NC group. **C.** The predicted miR-326 binding site of HOTAIR (HOTAIR-Wt) and the designed mutant sequence (HOTAIR-Mt) are indicated. Cells were transfected with HOTAIR-Wt (or HOTAIR-Mt) and the indicated miRNAs, and then the luciferase reporter assay was conducted. Data are presented as the mean ± SD (*n* = 5, each group). *P < 0.05 vs. HOTAIR-Wt + pre-NC group.

The expression of miR-326 in the human glioma tissues and cell lines were analyzed by real-time PCR. As shown in Figure 2A, miR-326 expression was significantly down-regulated in GT and two glioma cell lines compared with that in ST and NBTs, respectively. Furthermore, the miR-326 expression was negatively correlated with the histopathological grades of gliomas.

Emerging evidences have confirmed that lncRNAs might function as a competing endogenous RNA (ceRNA) or a molecular sponge in modulating miRNA [18, 20]. The possible miRNA binding site of HOTAIR was predicted by bioinformatics databases (Starbase v2.0). The miR-326 expression in stable sh-HOTAIR cell lines was evaluated, and results showed that the miR-326 expression was significantly up-regulated (Figure 2B). To further investigate whether HOTAIR was a functional target of miR-326, dual-luciferase reporter assay was performed. HOTAIR was predicted to harbor one miR-326 binding site. Our results showed that the luciferase activity was significantly decreased by the co-transfection of pre-miR-326 and HOTAIR-Wt rather than the co-transfection of pre-NC and HOTAIR-Wt, suggesting that HOTAIR was the target of miR-326. Meanwhile, co-transfection of pre-miR-326 and HOTAIR-Mt did not change the luciferase activity, suggesting that the miR-326 binding site within HOTAIR was functional (Figure 2C). Although the interaction between miR-326 and HOTAIR was confirmed, the biological behaviors of glioma cell regulated by miR-326 and HOTAIR need to be well confirmed.

### MiR-326 mediated the tumor-suppressive effects of HOTAIR knockdown on glioma cell lines

**Figure 3:**
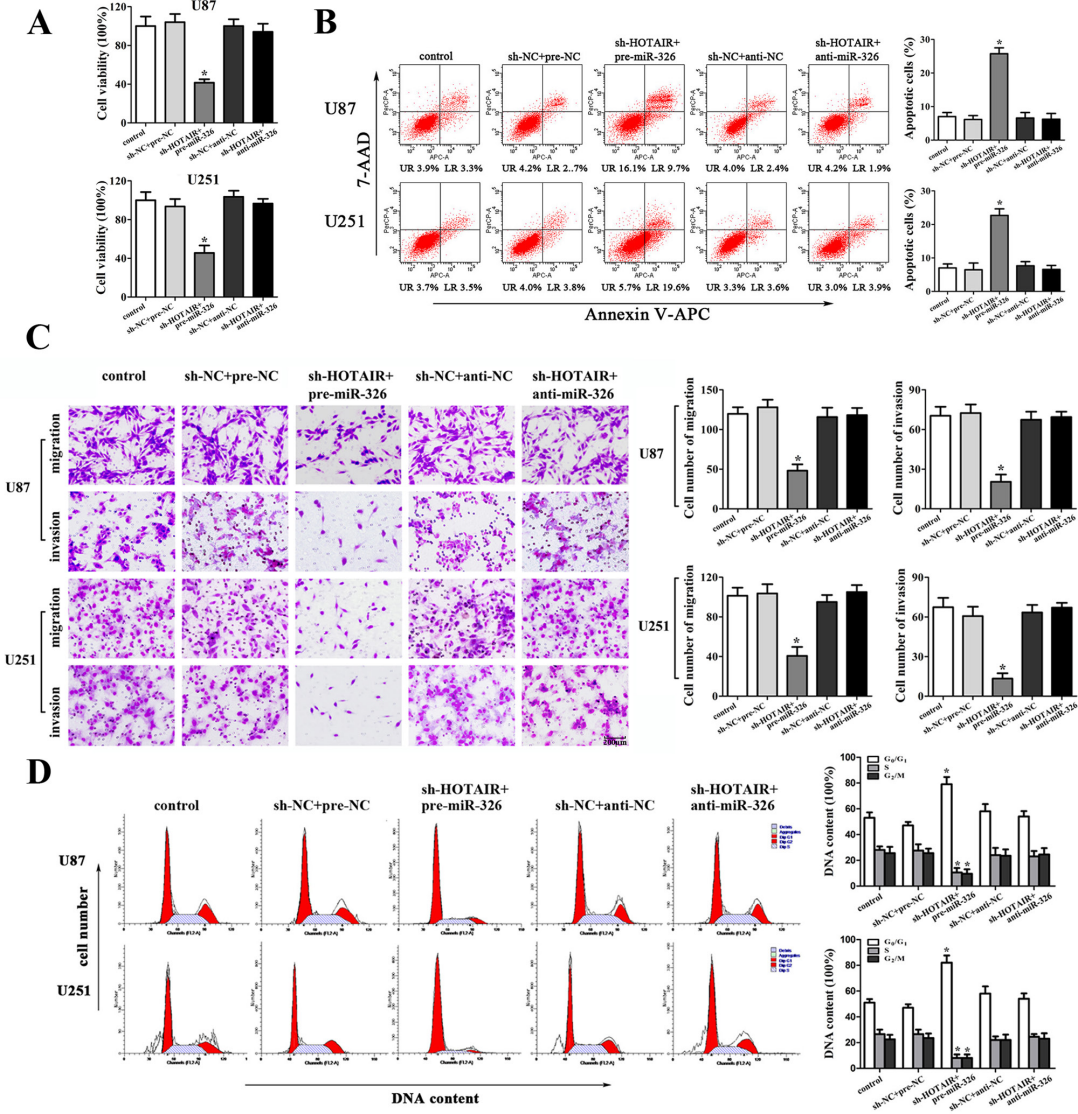
MiR-326 mediated the tumor-suppressive effects of HOTAIR knockdown on glioma cell lines **A.** CCK8 assay to evaluate the effect of HOTAIR and miR-326 on cell proliferation in U87 and U251 cells. **B.** Flow cytometry analysis to evaluate the effect of HOTAIR and miR-326 on cell apoptosis in U87 and U251 cells. **C.** Quantification of cells to evaluate the effect of HOTAIR and miR-326 on cell migration and invasion in U87 and U251 cells. Scale bars represent 200 μm. **D.** Flow cytometry analysis to evaluate the effect of HOTAIR and miR-326 on cell cycle in U87 and U251 cells. Data are presented as the mean ± SD (*n* = 5, each group). *P < 0.05 vs. sh-NC + pre-NC group.

To determine whether the tumor-suppressive effects of HOTAIR knockdown were mediated by miR-326, we transfected miR-326 mimics or miR-326 inhibitors into the stable sh-HOTAIR cells prior to the assessment of cell proliferation, apoptosis, migration, invasion and cell cycle. The stable sh-HOTAIR cells co-transfected with miR-326 mimics had the strongest inhibitory effect on cell proliferation, migration and invasion, and the highest apoptotic rate and the cell cycle arrest in G0/G1 phase. In addition, miR-326 inhibitors rescued the inhibitory effect of sh-HOTAIR on cell proliferation, migration and invasion, and miR-326 inhibitors rescued the increased apoptosis and cell cycle arrest in G0/G1 phase induced by sh-HOTAIR (Figure 3A, 3B, 3C and 3D). Based on the above results, we confirmed that miR-326 mediated the tumor-suppressive effects of HOTAIR knockdown on glioma cell lines. Furthermore, knockdown of HOTAIR combined with miR-326 over-expression enhanced the tumor-suppressive effects of HOTAIR knockdown on glioma cell lines.

### FGF1 was an oncogene in human glioma cells

**Figure 4:**
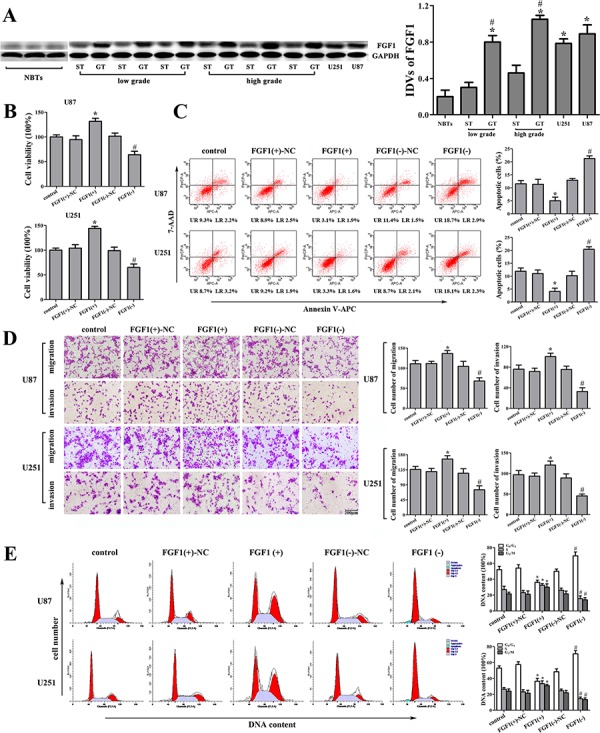
The effect of FGF1 on human glioma cell lines **A.** FGF1 expression in normal brain tissues (NBTs), different grades of human glioma tissues (GT), the surrounding non-neoplastic tissues (ST) and human glioma cell lines. Data are presented as the mean ± SD (*n* = 3, each group). *P < 0.01 vs. NBTs group. ^#^*P* < 0.05 vs. ST group. **B.** Effect of FGF1 on cell proliferation of U87 and U251 cells. **C.** Effect of FGF1 on apoptosis of U87 and U251 cells. **D.** Effect of FGF1 on cell migration and invasion of U87 and U251 cells. Scale bars represent 200 μm. **E.** Effect of FGF1 on cell cycle in U87 and U251 cells. Data are presented as the mean ± SD (*n* = 5, each group). *P < 0.05 vs. FGF1(+)-NC group, ^#^*P* < 0.05 vs. FGF1(−)-NC group.

FGF1 expression in human glioma tissues and cell lines was analyzed by Western blot. As shown in Figure 4A, FGF1 expression was significantly up-regulated in GT and two glioma cell lines compared with that in ST and NBTs, respectively. Furthermore, the FGF1 expression was positively correlated with the histopathological grades of glioma. We then explored the possible biological significance of FGF1 in glioma cells, and found that FGF1 over-expression promoted cell proliferation, migration and invasion, as well as inhibiting the cell apoptosis and cell cycle arrests in G0/G1 phase (Figure 4B, 4C, 4D and 4E). FGF1 knockdown exhibited the contrary effects. Therefore, FGF1 might be one of the factors that were responsible for the malignancy of glioma. However, whether FGF1 could be regulated by miR-326 and HOTAIR needed to be further investigated.

### Knockdown of HOTAIR inhibited the FGF1 expression by up-regulating miR-326

**Figure 5:**
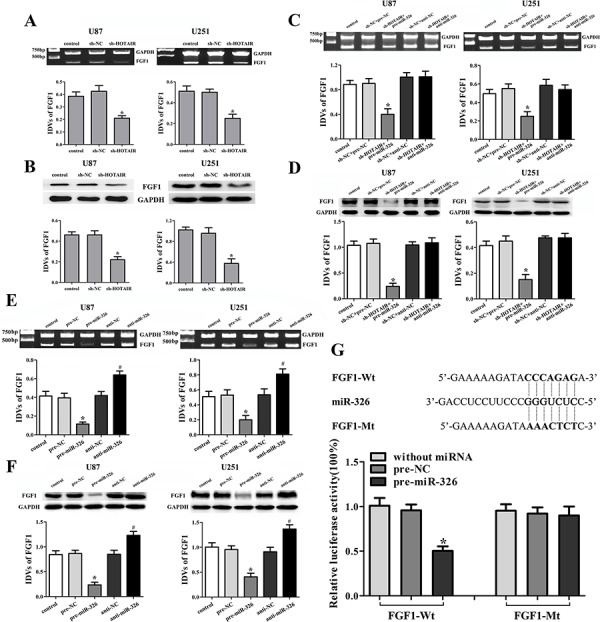
Knockdown of HOTAIR inhibited the FGF1 expression by up-regulating miR-326 Effects of HOTAIR knockdown on the expression of FGF1 in the mRNA **A.** and protein levels **B.** Data are presented as the mean ± SD (*n* = 5, each group). *P < 0.05 vs. sh-NC group. Effects of HOTAIR and miR-326 on the expression of FGF1 in the mRNA **C.** and protein levels **D.** Data are presented as the mean ± SD (*n* = 5, each group). *P < 0.05 vs. sh-NC + pre-NC group. Effects of miR-326 on the expression of FGF1 in the mRNA **E.** and protein levels **F.** Data are presented as the mean ± SD (*n* = 5, each group). *P < 0.05 vs. pre-NC group, ^#^*P* < 0.05 vs. anti-NC group. **G.** The predicted miR-326 binding sites in the 3′-UTR region of FGF1 (FGF1-Wt) and the designed mutant sequence (FGF1-Mt) are indicated. Cells were transfected with FGF1-Wt (or FGF1-Mt) and the indicated miRNAs, and then the Luciferase reporter assay was conducted. Data are presented as the mean ± SD (*n* = 5, each group). *P < 0.05 vs. FGF1-Wt + pre-NC group.

The expression levels of FGF1 were detected in the stable sh-HOTAIR U87 and U251 cell lines. Results showed that the knockdown of HOTAIR reduced the mRNA and protein expression levels of FGF1 (Figure 5A, 5B). In addition, in the sh-HOTAIR combined with miR-326 mimics group, the FGF1 expression was reduced in both mRNA and protein levels (Figure 5C, 5D). However, miR-326 inhibitors restored the low expression levels of FGF1 inducing by HOTAIR knockdown (Figure 5C, 5D). These results suggested that HOTAIR knockdown inhibited the FGF1 expression by up-regulating miR-326. In order to clarify the molecular mechanisms responsible for the inhibition of FGF1 expression, U87 and U251 cell lines were transfected with miR-326 mimics or inhibitors and the expression levels of FGF1 were detected. Results showed that miR-326 mimics down-regulated the FGF1 expression, while miR-326 inhibitors up-regulated it (Figure 5E, 5F). Furthermore, the luciferase reporter assay showed that the co-transfection of pre-miR-326 and FGF1-Wt strongly decreased the luciferase activity, but the co-transfection of pre-miR-326 and FGF1-Mt did not change it (Figure 5G). These results suggested that FGF1 was a direct target of miR-326.

### FGF1 mediated the tumor-suppressive effects of miR-326 over-expression on glioma cell lines

**Figure 6:**
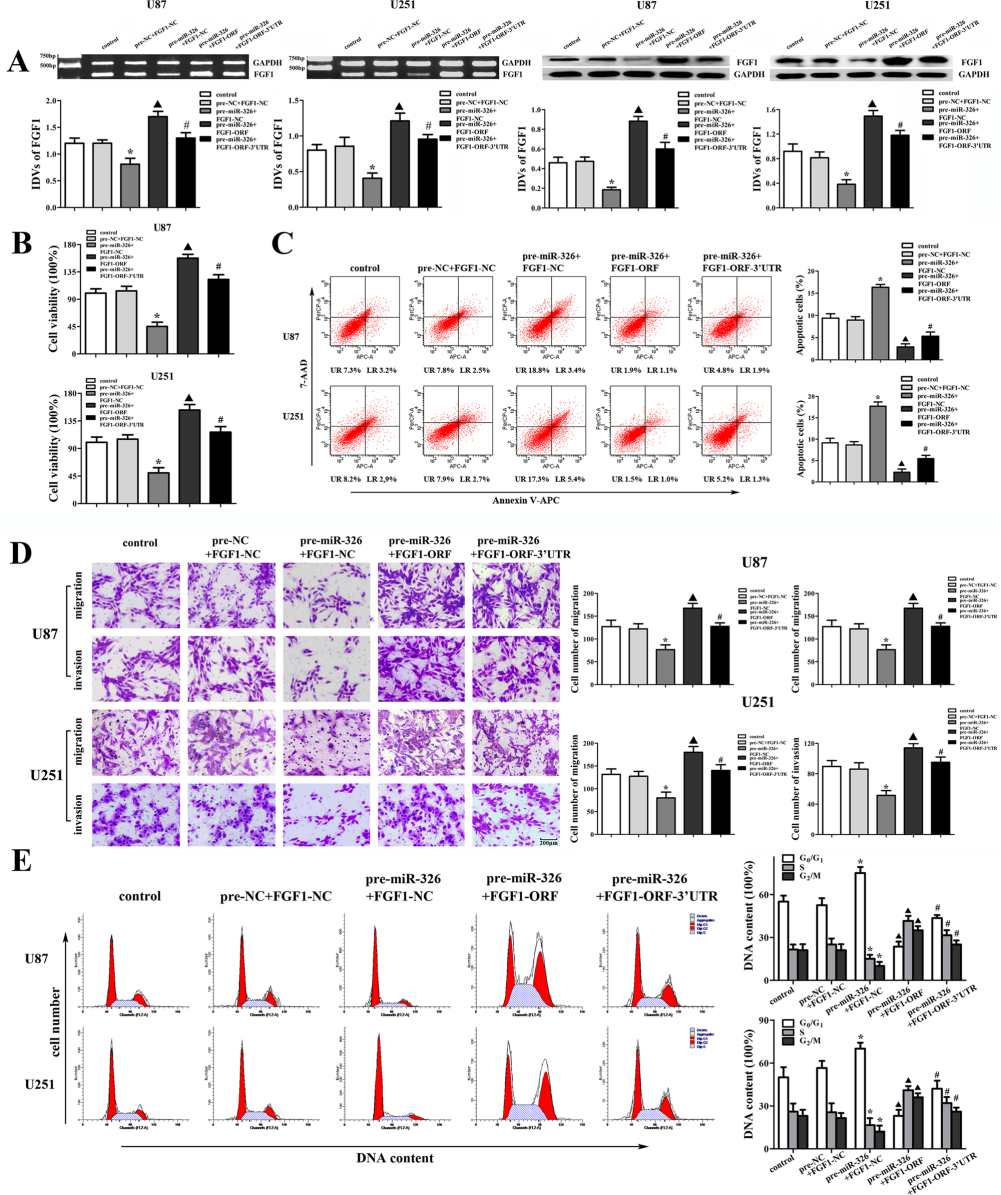
FGF1 mediated the tumor-suppressive effects of miR-326 over-expression on glioma cell lines **A.** The effects of miR-326 and FGF1-ORF (or FGF1-ORF-3′UTR) on the expression of FGF1 in the mRNA and protein levels in U87 and U251 cells. **B.** The effects of miR-326 and FGF1-ORF (or FGF1-ORF-3′UTR) on cell proliferation of U87 and U251 cells. **C.** The effects of miR-326 and FGF1-ORF (or FGF1-ORF-3′UTR) on apoptosis of U87 and U251 cells. **D.** The effects of miR-326 and FGF1-ORF (or FGF1- ORF-3′UTR) on cell migration and invasion of U87 and U251 cells. Scale bars represent 200 μm. **E.** The effects of miR-326 and FGF1-ORF (or FGF1-ORF-3′UTR) on the cell cycle in U87 and U251 cells. Data are presented as the mean ± SD (*n* = 5, each group). *P < 0.05 vs. pre-NC + FGF1-NC group, ^▲^*P* < 0.05 vs. pre-miR-326 + FGF1-NC group, ^#^*P* < 0.05 vs. pre-miR-326 + FGF1-ORF group.

To determine whether the tumor-suppressive effects of miR-326 were mediated by FGF1, down-regulated FGF1 by miR-326 mimics was rescued using FGF1 prior to the assessment of the cell proliferation, apoptosis, migration, invasion and cell cycle. As shown in Figure 6A, over-expressed miR-326 inhibited FGF1 expression both in mRNA and protein levels, and FGF1-ORF recued the inhibitory effect induced by miR-326 over-expression. However, FGF1-ORF-3′UTR did not recued these effects. These results suggested that miR-326 inhibited the FGF1 expression by targeting its 3′-UTR in U87 and U251 glioma cells. The following experiments showed that miR-326 inhibited the proliferation, migration and invasion, induced the apoptosis and cell cycle arrest in G0/G1 phase by targeting the 3′-UTR of FGF1 (Figure 6B, 6C, 6D and 6E). These results indicated that miR-326 played a tumor-suppressive role in glioma by targeting the 3′-UTR of FGF1.

### FGF1 activated the PI3K/AKT and MEK1/2 signal pathways

**Figure 7:**
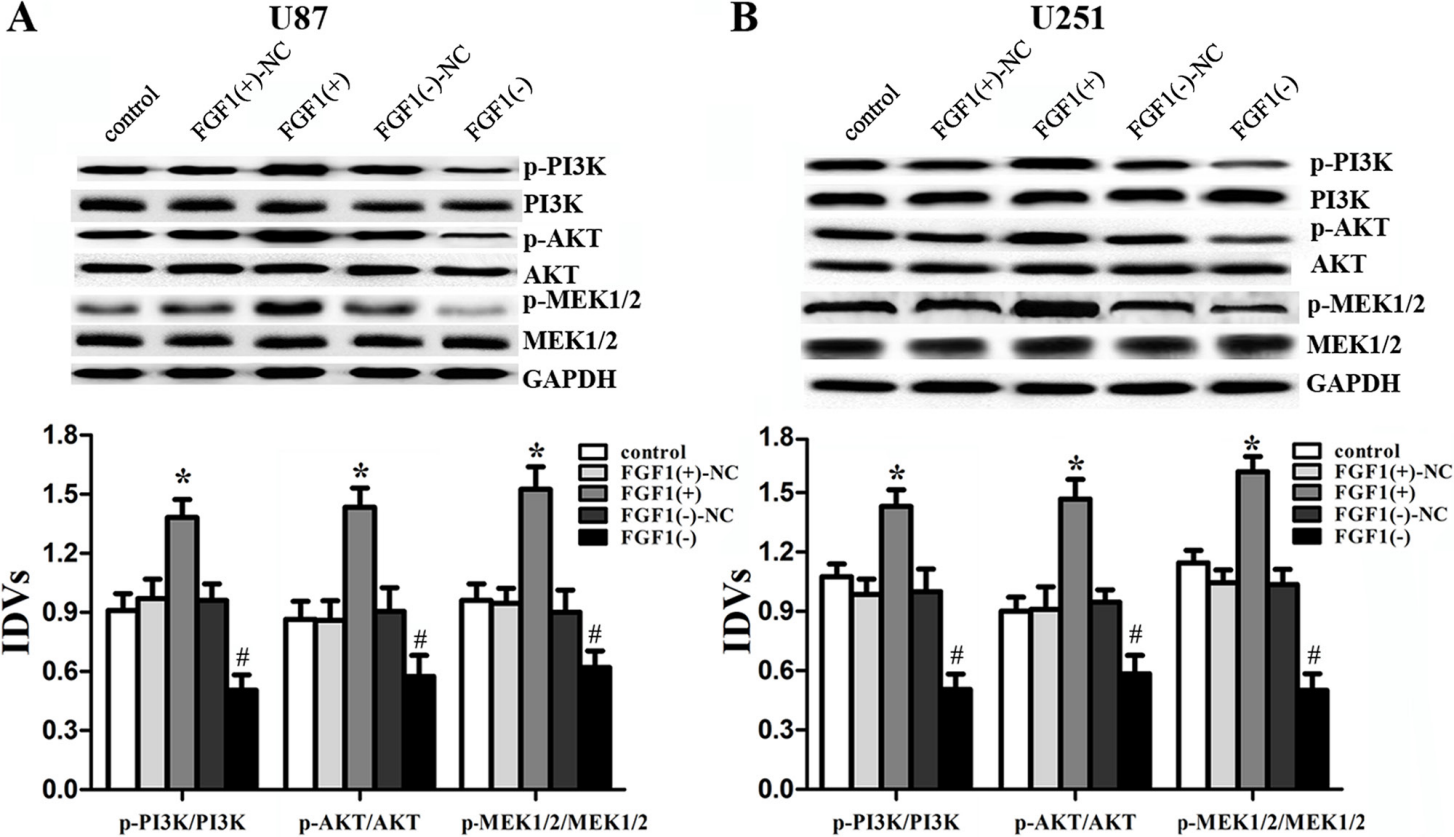
FGF1 activated the PI3K/AKT and MEK1/2 signal pathways Western blot analysis of the PI3K/AKT and MEK 1/2 pathways regulated by FGF1 in U87 **A.** and U251 **B.** cells. Data are presented as the mean ± SD (*n* = 5, each group). *P < 0.05 vs. FGF1(+)-NC group, ^#^*P* < 0.05 vs. FGF1(−)-NC group.

To figure out the molecular mechanisms of FGF1 oncogenic functions, the activities of PI3K/AKT and MEK1/2 signal pathways were detected in the glioma cell lines. Over-expressed FGF1 activated the PI3K/AKT and MEK1/2 signal pathways, while FGF1 knockdown inhibited these pathways (Figure 7A, 7B). These results confirmed that PI3K/AKT and MEK 1/2 signal pathways were involved in the FGF1 oncogenic functions.

### Knockdown of HOTAIR combined with miR-326 over-expression suppressed tumor growth and prolonged the survival of nude mice

**Figure 8:**
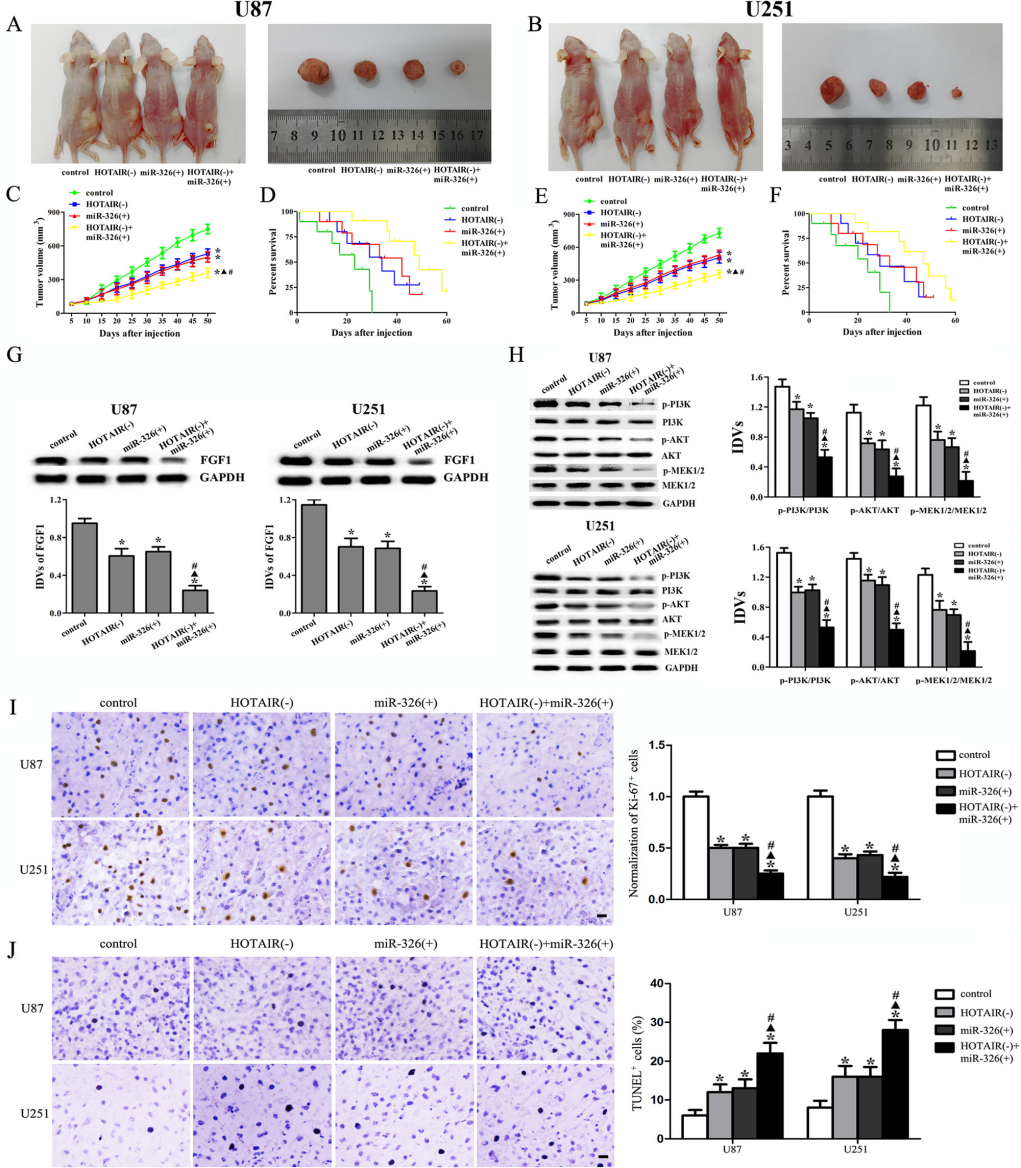
*In vivo* tumor xenografts study The stable expressing cells were used for the *in vivo* study. **A and B.** The nude mice carrying tumors and the sample tumor from respective groups were shown. **C and E.** Tumor growth curves in nude mice. Tumor volume was calculated every five days after injection. **D and F.** The survival curves of nude mice injected into the right striatum (*n* = 15). **G.** FGF1 expression from respective group. **H.** The activities of PI3K/AKT and MEK 1/2 pathways from respective group. **I.** Representative staining of Ki-67 (left) and quantification of relative fraction of Ki-67^+^ cells (right) are shown. **J.** Representative TUNEL staining (left) and quantification of fraction of TUNEL^+^ cells are shown (right). Data are presented as the mean ± SD (*n* = 5, each group). *P < 0.05 vs. control group, ^▲^*P* < 0.05 vs. HOTAIR(−) group, ^#^*P* < 0.05 vs. miR-326(+) group.

As shown in Figure 8A, 8B, 8C and 8E, the tumor volume was smallest in the group of HOTAIR knockdown combined with miR-326 over-expression. In the HOTAIR knockdown group or the miR-326 over-expression group, the tumor volume was smaller than that in the control group, whereas the tumor volume was larger than that in the combined group. As shown in Figure 8D and 8F, the combined group had the highest survival rates. The HOTAIR knockdown group or the miR-326 over-expression group had higher survival rates than the control group, whereas the survival rates in these two groups were lower than those in the combined group. These results suggested that knockdown of HOTAIR combined with miR-326 over-expression could suppress the tumor growth and prolonged the survival in nude mice.

As shown in Figure 8G and 8H, the expression levels of FGF1, p-PI3K/PI3K, p-AKT/AKT and p-MEK1/2/MEK1/2 were the lowest in the combined group and were lower in the HOTAIR knockdown or miR-326 over-expression group than those in the control group, whereas they were higher than those in the combined group. Furthermore, we examined the expression of Ki-67 to investigate proliferative activity and the apoptosis in tumors by TUNEL assay. As shown in Figure 8I, the number of proliferative Ki-67 positive cells was least in the HOTAIR knockdown combined with miR-326 over-expression group. The number of actively proliferative Ki-67 positive cells in the HOTAIR knockdown group or the miR-326 over-expression group was less than the control group but more than the combined group. Furthermore, TUNEL analysis revealed that the combined group exhibited the highest rates of cell apoptosis. The apoptosis rate in the HOTAIR knockdown or miR-326 over-expression group was higher than that in the control group, whereas the apoptosis rate was lower than that in the combined group (Figure 8J).

## DISCUSSION

In this study, we demonstrated that HOTAIR was highly expressed in the glioma tissues and cell lines. Knockdown of HOTAIR could suppress the malignant behaviors of glioma cells. In addition, the HOTAIR knockdown could up-regulate miR-326 that were lowly expressed in the glioma tissues and cell lines. The further study showed that miR-326 played a tumor-suppressive role by down-regulating FGF1 in glioma cell. The mechanism study showed that PI3K/AKT and MEK 1/2 signal pathways were involved in this progress. The *in vivo* study also demonstrated that knockdown of HOTAIR combined with miR-326 over-expression produced the smallest tumor and the longest survival in nude mice. Taken together, the HOTAIR-miR-326-FGF1 axis played an important role in human glioma and suggested a promising therapeutic target in glioma treatment.

Our present study showed that HOTAIR expression was up-regulated in human glioma tissues and cell lines, suggesting that HOTAIR may be critically involved in the development of glioma. Furthermore, we confirmed that the knockdown of HOTAIR inhibited the proliferation, migration and invasion, promoted the apoptosis and induced the cell cycle arrest in G0/G1 phase in glioma cells, indicating that HOTAIR played an oncogenic role in human glioma. Consistent with our results, HOTAIR is over-expressed in early and metastatic breast cancer cells [21], serves as a prognostic factor for glioma patient [13] and promotes glioblastoma cell cycle progression in an EZH2 dependent manner [14]. Our findings provided solid evidence for the HOTAIR functional role in human glioma. Previous study have shown that HOTAIR could alter histone H3 lysine 27 methylation, and increase cancer invasion and metastasis in a manner dependent on PRC2 by form a complex with Polycomb repressive complex 2 (PRC2) [21–23]. We further focused on the direct target of HOTAIR.

Our results showed that miR-326 had a low copy number in human glioma tissues and cell lines. Previous studies have also shown the low expression levels of miR-326 in several cancers including colorectal cancer [24], multiple sclerosis [25] and glioblastoma [26]. Furthermore, miR-326 also acts as a prognostic indicator in human glioma [27]. Emerging evidence have confirmed that lncRNAs might function as a competing endogenous RNA (ceRNA) or a molecular sponge in modulating miRNA [18, 20]. HOTAIR functions as a competing endogenous RNA to regulate HER2 expression by sponging miR-331-3p in gastric cancer [18], and is targeted and regulated by miR-141 in human renal carcinoma cells [19]. In addition, estradiol induces HOTAIR expression through the suppression of miR-148a in breast cancer [28]. We used the bioinformatics databases (Starbase v2.0) to predict the miRNA binding site of HOTAIR. Meanwhile, our present study showed that knockdown of HOTAIR increased miR-326 expression. Furthermore, HOTAIR was identified as the target of miR-326, and miR-326 mediated the tumor-suppressive effects of HOTAIR knockdown on glioma cell lines. HOTAIR knockdown combined with miR-326 over-expression suppressed the tumor growth and had high survival rates in nude mice. Based on the above results, HOTAIR knockdown combined with miR-326 over-expression exerted the tumor-suppressive function *in vivo* and *in vitro*, and there was a negative interaction between these two factors. However, the functional role of miR-326 in this progress was still unknown. Previous study showed that miR-326 acted as a tumor-suppressive factor by down-regulating pyruvate kinase type M2 (PKM2) [26] and Nin one binding protein (NOB1) in human glioma [29]. Additionally, miR-326 inhibited the biological behaviors and stemness of glioma cells by targeting smoothened (SMO) [30]. Thus, miR-326 might have a downstream target that could be regulated by miR-326.

The fibroblast growth factor-1 (FGF1), a member of fibroblast family, could promote the repairing progress of vessel damaged [31]. In particular, members of FGF family have been shown to contribute to the tumor cell proliferation and resistance to chemotherapy in human pancreatic cancer cell lines [32, 33]. The high copy numbers of FGF1 have been found in prostate [34], non-small-cell lung [35], breast [36] and ovarian cancers [37]. In our present study, the FGF1 expression was up-regulated in human glioma tissues and cell lines. Furthermore, our study showed that FGF1 played an oncogenic role in human glioma cells. However, whether FGF1 was involved in the tumor-suppressive effects of HOTAIR knockdown needed to be further studied. Recent studies showed that miR-195 could suppress the expression of FGF1 in vascular smooth muscle cells [38], and miR-9 targeted FGF1 in midbrain-hindbrain boundary [39]. Our results showed that knockdown of HOTAIR resulted in the low mRNA and protein expression levels of FGF1. Moreover, miR-326 over-expression enhanced the effects of low FGF1 expression induced by HOTAIR knockdown and miR-326 inhibition reversed the above effects. These results indicated that the knockdown of HOTAIR inhibited the FGF1 expression by up-regulating miR-326, and the luciferase reporter assay confirmed that FGF1 was the direct target of miR-326. Our results further demonstrated that miR-326 inhibited the proliferation, migration and invasion, induced the apoptosis and cell cycle arrest in G0/G1 phase by targeting the 3′-UTR of FGF1 in human glioma cells.

**Figure 9:**
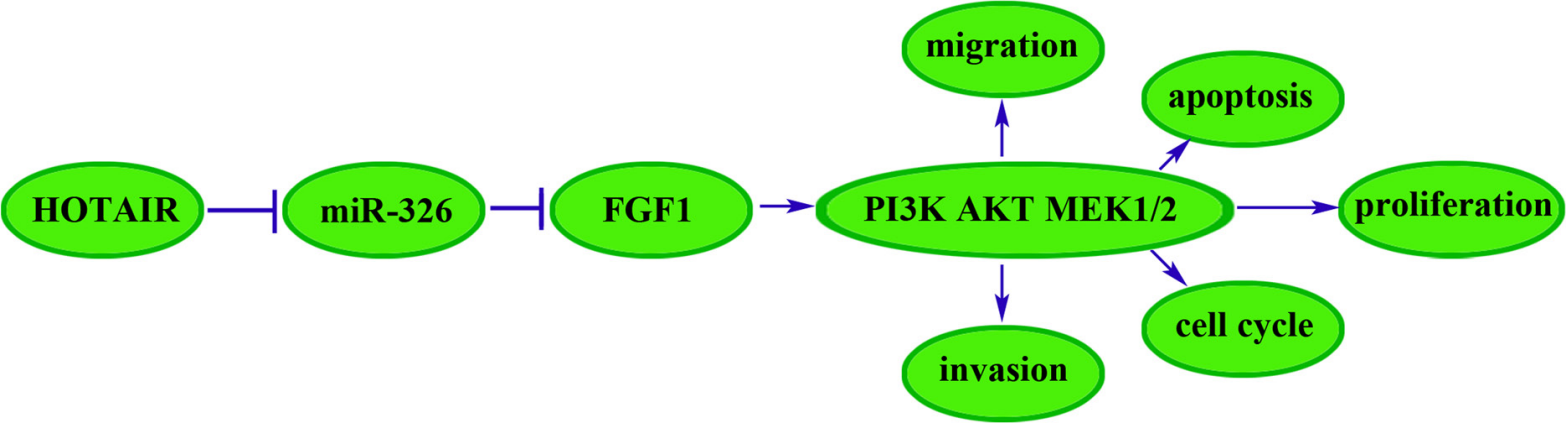
The cartoon of the mechanism underlying the HOTAIR-miR-326-FGF1 axis in U87 and U251 cells

Taken together, we found that the HOTAIR-miR-326-FGF1 axis had an important role in human glioma. In brief, the miR-326 over-expression induced by the knockdown of HOTAIR could down-regulate the FGF1 expression that acted as an oncogenic factor in human glioma. We further investigated the molecular mechanisms of FGF1 oncogenic functions. Previous studies showed that FGF family members could target the fibroblast growth factor receptor (FGFR) to promote the tumor growth, angiogenesis and metastasis by regulating tumor cells, endothelial cells and pericytes [40, 41]. The downstream pathways of FGFR, including MAPK, PI3K, Ras and JNK pathways, could promote tumor growth and metastasis, and take part in every step of the tumor formation [42–46]. In A549 cells, FGF1 induced FGFR phosphorylation, leading to the activation of JNK pathway [35]. And FGF1 induced FGFR phosphorylation to activate PI3K and JNK, resulting in the activation of MMP7 and MMP6 in hepatocellular carcinoma, which might be the underlying mechanism in the hepatocellular carcinoma metastasis [47]. In this work, we showed that FGF1 over-expression activated the PI3K/AKT and MEK 1/2 pathways in U87 and U251 cell lines, and knockdown of FGF1 exerted the opposite effects. Therefore, the reduced FGF1 induced by miR-326 over-expression could attenuate the activity of PI3K/AKT and MEK 1/2 pathways to inhibit the malignant behaviors of glioma cells. In addition, the *in vivo* studies also supported the above findings. The mechanism underlying tumor suppressive function of human glioma cells by HOTAIR knockdown is schematically presented in Figure 9.

In conclusion, our study revealed that knockdown of HOTAIR inhibited the proliferation, cell migration and invasion, and promoted apoptosis and cell cycle arrest by up-regulating miR-326 in human glioma cells. Over-expression of miR-326 inhibited FGF1 expression by targeting its 3′-UTR and further blocked the activity of PI3K/AKT and MEK1/2 pathways, leading to the inhibition of malignant behaviors of glioma cells. Therefore, the HOTAIR-miR-326-FGF1 axis might represent a promising therapeutic strategy for the treatment of human glioma.

## MATERIALS AND METHODS

### Human tissue samples

All the tumor tissue samples were collected from patients who were undergoing complete or partial surgical resection at the Department of Neurosurgery, Shengjing Hospital of China Medical University during 2013–2014. The tissue samples were obtained from the treatment naive patients. Glioma samples were divided into two groups: low grade (grade I–II) and high grade (grade III–IV) according to the WHO classification. The diagnosis of glioma was established histologically by two experienced clinical pathologists according to the WHO classification. Informed consent was obtained from all patients and this study procedure was approved by Research Ethics Board at the Shengjing Hospital of China Medical University.

### Cell culture

The glioma cell lines (U87 and U251) and human embryonic kidney (HEK) 293T cells were obtained from Shanghai Institutes for Biological Sciences Cell Resource Center and were cultured in high glucose DMEM supplemented with 10% fetal bovine serum (Gibco, Carlsbad, CA, USA). All the cells were incubated at 37°C in a humidified incubator with 5% CO_2_.

### RNA extraction and real-time PCR

Total RNA was extracted from cells and tissues using Trizol reagent (Life Technologies Corporation, Carlsbad, CA, USA) according to the manufacturer's instructions. The One Step SYBR^®^ PrimeScript^®^PLUS RT-RNA PCR Kit (TaKaRa Biotechnology, Dalian, China) was used for the Real-Time PCR analysis to test the expression levels of HOTAIR. The primers are: forward: 5′-GGCAAATGTCAGAGGGTT-3′; reverse: 5′-GTGTA ACAGGCAGGTGGA-3′. And the Taqman MicroRNA Reverse Transcription Kit and Taqman Universal Master Mix II with the TaqMan MicroRNA Assay of miR-326 and U6 (Applied Biosystems, Foster City, CA, USA) were used for testing the expression levels of miR-326 in cells and tissues. For the test of FGF1, RNA PCR Kit (AMV) Ver.3.0 (TaKaRa Biotechnology, Dalian, China) was used, and the primers are: forward: 5′-AGGTTGGTAGTTCCCTGCTCT-3′; reverse: 5′-GGTT CCTTTGCCTCTGACAC-3′. The GAPDH used in this study for normalizing and the primers are: forward: 5′-CGCTGAGTACGTCGTGGAGT-3′; reverse: 5′-CGTC AAAGGTGGAGGAGTGG-3′. Fold changes were calculated by relative quantification (2^−ΔΔCt^) method.

### Transfection and generation of stably transfected cell lines

Short-hairpin RNA directed against human lncRNA HOTAIR was ligated into the U6/GFP/Neo plasmid (GenePharma, Shanghai, China) and was referred as to sh-HOTAIR. For the analysis of the FGF1 functions, the full-length FGF1 with 3′-UTR sequences and short-hairpin RNA directed against FGF1 were constructed in pEX-2 and U6/GFP/Neo plasmids (GenePharma), respectively. And they were referred as to FGF1(+) and FGF1(−). For analysis of the mechanism of FGF1 regulated by miR-326, the full-length FGF1 with 3′-UTR sequences (or without 3′-UTR sequences) was constructed in pEX-2 plasmids (GenePharma) and were referred as to FGF1-ORF-3′UTR (or FGF1-ORF). The lipofectamine 2000 reagent (Life Technologies Corporation, Carlsbad, CA, USA) was used for the cells transfection according to the manufacturer's instructions. The plasmid carrying a non-targeting sequence was used as a negative control (NC) of sh-HOTAIR and FGF1(−) which were referred as to sh-NC and FGF1(−)-NC. The empty plasmid was used as NC of FGF1(+) (or FGF1-ORF-3′UTR, FGF1-ORF) which was referred as to FGF1(+)-NC (or FGF1-NC). The stably transfected cells were selected by the culture medium containing 0.5 mg/ml G418 (Sigma-Aldrich, St Louis, MO, USA). After approximately 4 weeks, G418-resistant cell clones were established. MiR-326 mimics, inhibitors and their respective NC were synthesized (Life Technologies Corporation, MD, USA) and transfected into cells in the *in vitro* study. Because the highest transfection efficiency was occurred at 48 h, thus 72 h post-transfection was considered as the harvest time in the subsequent experiments.

### Cell proliferation assay

Cell proliferation assays were performed using the Cell Counting Kit-8 (CCK8, Beyotime Institute of Biotechnology, Jiangsu, China) according to the manufacturer's instructions. Cells were seeded into 96-well cell culture plates with five replicate wells for each group, and assayed at 72 h after transfection. CCK8 was added into each well and incubated for another 4 h, and the absorbance was finally measured at the wavelength of 450 nm.

### Quantization of apoptosis by flow cytometry

The cells were harvested and were stained with Annexin V-APC/7-AAD (KeyGEN Biotech, Nanjing, China) according to the instruction of the manufacturer. Then the cells were acquired by flow cytometry (FACScan, BD Biosciences, USA) and analyzed by CELL Quest 3.0 software.

### Cell migration and invasion assay

The abilities of cell migration and invasion were tested using the 24-well transwell chambers with 8 μm pore size polycarbonate membrane (Corning, NY, USA) accroding to the manufacturer's protocol. The harvested cells were re-suspended in serum-free medium then seeded in the upper chamber on the top side of membrane, or the upper chambers were pre-coated with Matrigel solution (BD, Franklin Lakes, NJ, USA) and incubated at 37°C for 4 h before the invasion assay started. Migrated and invaded cells on the lower membrane surface were fixed with methanol and glacial acetic acid and then stained with 20% Giemsa solution. Five randomly fields were counted randomly in each well.

### Cell cycle analysis

The transfected cells were harvested and then fixed with 500 μl of 70% cold ethanol for 2 h. The cells were added with 100 μl of RNase and incubated at 37°C for 30 min. Then, 400 μl of PI was added, and the cells were incubated at 4°C for 30 min away from light. The samples were immediately subjected to flow cytometer (FACScan, BD Biosciences, USA). The results were analyzed using CELL Quest 3.0 software.

### Reporter vectors constructs and luciferase reporter assay

The fragment from HOTAIR containing the predicted miR-326 binding site was amplified by PCR and then cloned into a pmirGlO Dual-luciferase miRNA Target Expression Vector (Promega, Madison, WI, USA) to form the reporter vector HOTAIR-wild-type (HOTAIR-Wt). To mutate the putative binding site of miR-326 in the HOTAIR, the sequence of putative binding site was replaced as indicated and was named as HOTAIR-mutated-type (HOTAIR-Mt). Similarly, the fragment from FGF1 3′-UTR sequences were amplified by PCR and cloned into a pmirGlo Dual-luciferase miRNA Target Expression Vector to form the reporter vector FGF1-wild-type (FGF1-Wt) (GenePharma). To mutate the putative binding site of miR-326 in the 3′-UTR-containing vector, the sequence of putative binding site was replaced as indicated and was named as FGF1-mutated-type (FGF1-Mt). Then the vectors and miR-326 mimics were co-transfected into HEK 293T cells, and the Dual-Luciferase Reporter Assay System (Promega, Madison, WI, USA) were used for testing the luciferase activity.

### Western blot assay

Total protein of cells were lysed using ice-cold RIPA buffer supplemented with protease inhibitors and centrifuged at 14, 000 × g 4°C for 5 min. 10 μg of total protein was used for Western blot analysis. Expression of protein was confirmed by FGF1 (rabbit, polyclonal, IgG1, 1:500, Santa Cruz Biotechnology, CA, USA); PI3K, p-PI3K (rabbit, monoclonal, IgG1, 1:1000, Santa Cruz Biotechnology, CA, USA); AKT, p-AKT (rabbit, monoclonal, IgG1, 1:1000, Santa Cruz Biotechnology, CA, USA); MEK 1/2, p-MEK 1/2 (rabbit, monoclonal, IgG1, 1:1000, Santa Cruz Biotechnology, CA, USA); and GAPDH (mouse, monoclonal, IgG1, 1:1000, Santa Cruz Biotechnology, CA, USA), followed by incubation with appropriate correlated HRP-conjugated secondary antibody. Immunoblots were visualized by enhanced chemiluminescence (ECL kit, Santa Cruz Biotechnology, CA, USA) and scanned using ChemImager 5500 V2.03 software. The relative integrated density values (IDVs) were calculated by Fluorchem 2.0 software and normalized with GAPDH.

### *In vivo* studies

For the *in vivo* study, the stably transfected cell lines were used. The plasmid pGCMV/EGFP/miR-326 (GenePharma) was transfected into cells and selected by the culture medium containing 10 μg/ml Blasticidin (Life Technologies Corporation, Carlsbad, CA, USA) to generate miR-326 (+) stably transfected cell lines. To generate HOTAIR(−) + miR-326(+) stably transfected cell lines, the pGCMV/EGFP/miR-326 plasmids was transfected in HOTAIR (−) stably transfected cells and selected by the culture medium containing 10 μg/ml Blasticidin.

The 4-week old BALB/C athymic nude mice were obtained from Cancer Institute of the Chinese Academy of Medical Science. Experiments with mice were conducted strictly in accordance with a protocol approved by the Administrative Panel on Laboratory Animal Care of the Shengjing Hospital. For subcutaneous implantation, 3 × 10^5^ cells were injected subcutaneously into the flank area of each animal. The animals were weighed and the tumors were measured every five days, until 42 days post-injection. Tumor volume was calculated according to the following formula: tumor volumes (mm^3^) = length × width^2^/2. For orthotopic tumor inoculations, 3 × 10^5^ cells were injected into the right striatum of the nude mice. Mice were sacrificed by CO_2_ inhalation and death was confirmed by cervical dislocation if they exhibited excessive weight loss of 20% body weight, tumor metastasis, lethargy, or other signs of distress consisted with IACUC standards. The number of survived nude mice was recorded every 5 days, until 60 days.

### Immunohistochemistry

Tumors from subcutaneous implantation assay were fixed in 4% paraformaldehyde, and then were dehydrated, embedded in paraffin, and cut. Consecutive 4 μm thick sections were analyzed by immunohistochemistry using antibodies against Ki-67 (Bioworld Technology, Louis Park, MN, USA). Antigen retrieval was performed using citrate buffer at pH 6.0. DAB (Beyotime Institute of Biotechnology, Jiangsu, China) systems were used for detection.

### TUNEL assay

TUNEL assay was performed using the Colorimetric TUNEL Apoptosis Assay Kit (Beyotime Institute of Biotechnology, Jiangsu, China) in accordance with the manufacturer's protocol. The sections were incubated in 3% H_2_O_2_ and incubated in the TUNEL reaction mixture. The sections were rinsed and visualized using DAB. Hematoxylin was used for counter-staining. The number of TUNEL-positive cells was counted in six fields randomly, and the apoptosis index for each field was calculated as the percent of TUNEL-positive cells relative to the total cells.

### Statistical analysis

Experimental data were expressed as means ± standard deviation (SD). The *t* test or one-way analysis of variance (ANOVA) was used to determine statistical significance. All statistical analyses were performed using SPSS 18.0 statistical software considering *P* < 0.05 as statistically significant. Corresponding significance levels are indicated in the figures.
